# Cut‐offs for calf circumference as a screening tool for low muscle mass: WASEDA'S Health Study

**DOI:** 10.1111/ggi.14025

**Published:** 2020-09-04

**Authors:** Ryoko Kawakami, Motohiko Miyachi, Susumu S. Sawada, Suguru Torii, Taishi Midorikawa, Kumpei Tanisawa, Tomoko Ito, Chiyoko Usui, Kaori Ishii, Katsuhiko Suzuki, Shizuo Sakamoto, Mitsuru Higuchi, Isao Muraoka, Koichiro Oka

**Affiliations:** ^1^ Faculty of Sport Sciences Waseda University Tokorozawa Japan; ^2^ Department of Physical Activity Research, National Institutes of Biomedical Innovation, Health and Nutrition Tokyo Japan; ^3^ College of Health and Welfare, J. F. Oberlin University Tokyo Japan

**Keywords:** anthropometry, bioelectrical impedance, body composition, dual‐energy X‐ray absorptiometry scan, sarcopenia

## Abstract

**Aim:**

To re‐evaluate the suitability of calf circumference as a surrogate marker of low muscle mass measured by both bioelectrical impedance analysis (BIA) and dual‐energy X‐ray absorptiometry (DXA). We also examined the effects of obesity and age on low muscle mass screening using calf circumference.

**Methods:**

In total, 1239 adults participated in this cross‐sectional study. We measured the maximum calf circumference in a standing position and appendicular skeletal muscle mass (ASM) using BIA and DXA. We defined low muscle mass based on the Asian Working Group for Sarcopenia 2019 consensus.

**Results:**

Calf circumference was positively correlated with BIA‐measured ASM/height^2^ (men: *r* = 0.81, women: *r* = 0.73) and DXA‐measured ASM/height^2^ (men: *r* = 0.78, women: *r* = 0.76). In the subgroup analyses by obesity and age, calf circumference was also positively correlated with ASM/height^2^. The optimal calf circumference cut‐offs for low muscle mass screening measured by BIA and DXA were 35 cm (sensitivity 91%, specificity 84%) and 36 cm (sensitivity 82%, specificity 80%) for men, and 33 cm (sensitivity 82%, specificity 84%) and 34 cm (sensitivity 85%, specificity 72%) for women, respectively.

**Conclusions:**

Calf circumference is positively correlated with BIA‐ and DXA‐measured muscle mass regardless of obesity and age and is a simple and accurate surrogate marker of muscle mass for diagnosing sarcopenia. **Geriatr Gerontol Int 2020; 20: 943–950**.

## Introduction

Sarcopenia,^1^ defined as age‐related loss of muscle mass and function, is associated with a higher risk of falls, fractures^2^ and mortality.^3^ Loss of muscle mass starts in middle adulthood^4^ and must be detected during the early stages because its effects can be prevented by lifestyle interventions such as resistance training.^5^

Dual‐energy X‐ray absorptiometry (DXA) is most widely used for assessing muscle mass in the diagnosis of sarcopenia.^1^ However, DXA is not suitable for assessments aimed at prevention because the device used for DXA is expensive, non‐portable and exposes participants to radiation. The Asian Working Group for Sarcopenia (AWGS) revised the consensus on sarcopenia diagnosis and treatment for Asian individuals and proposed calf circumference as a case‐finding tool for primary healthcare and community‐based health promotion.^6^ Calf circumference is suitable for the simple assessment of muscle mass in clinical settings because it is easy to measure.

Calf circumference is positively correlated with appendicular skeletal muscle mass (ASM) and ASM/height^2^ measured by DXA,^7^, ^8^, ^9^, ^10^, ^11^, ^12^ suggesting that calf circumference can be used as a screening tool for low muscle mass (LMM). Calf circumference cut‐offs of <34 and <33 cm for men and women, respectively, have been proposed by the AWGS 2019 consensus for finding cases of sarcopenia.^6^ However, few studies have examined the optimal calf circumference cut‐offs for LMM screening in Asian populations.^7^, ^8^, ^11^, ^13^, ^14^ Cut‐offs for ASM/height^2^ for determining LMM using bioelectrical impedance analysis (BIA) and DXA are separately proposed by the AWGS 2019 consensus.^6^ However, whether the calf circumference cut‐offs are consistent with LMM measured by either BIA or DXA remains unknown. Moreover, body fat mass affects morphometric indicators, and the impact of obesity and age on LMM screening measured by calf circumference is unknown.

Therefore, we aimed to investigate the relationship between calf circumference and ASM/height^2^ measured by both BIA and DXA and to re‐evaluate the validity of the optimal calf circumference cut‐offs for LMM screening. Furthermore, we examined the effect of obesity and age on LMM screening measured by calf circumference.

## Methods

### *Participants*

This cross‐sectional study used baseline measurements from the Waseda Alumni's Sports, Exercise, Daily Activity, Sedentariness and Health Study (WASEDA'S Health Study), a prospective cohort study whose participants included Waseda University alumni and their spouses aged ≥40 years. The participants selected one of four cohorts (cohorts A–D) with different measurement items. The study participants comprised 1296 middle‐aged and elderly adults who received calf circumference, muscle strength, BIA and DXA measurements in cohort D of the WASEDA'S Health Study between March 2015 and January 2020. Of the 1296 participants, we excluded foreign nationals (*n* = 6), those with metal implants or fragments in their bodies and those unable to remove metal items they were wearing at the time of measurement (*n* = 51), leaving 1239 participants (827 men and 412 women) for analysis.

The study was approved by the Research Ethics Committee of Waseda University and conducted in accordance with the Declaration of Helsinki (approval number: 2014‐G002). All participants received an explanation of the study before measurement and provided their written informed consent.

### *Anthropometric measurements*

Trained researchers measured all participants in the morning after the participants had fasted for ≥12 h, and recorded height and weight in 0.1‐cm and 0.1‐kg increments, respectively, and calf circumference in 0.1‐cm increments using a steel measuring tape (F10‐02DM; Muratec‐KDS Corp., Kyoto, Japan). Calf circumference was measured according to the protocol of the International Society for the Advancement of Kinanthropometry. Care was taken not to compress the subcutaneous tissue when placing the measuring tape around the calf and when measuring the calf circumference twice on each side where the circumference was the largest in the standing position; the average was calculated (Fig. S1).

### *Muscle mass measurements*

We used multifrequency BIA analyzer (MC‐980A; Tanita Corp., Tokyo, Japan) to measure bioimpedance according to the manufacturer's protocol, with participants wearing light clothing and barefoot. The device uses six electric frequencies (1, 5, 50, 250, 500 and 1000  kHz) and employs the manufacturer's proprietary formula to estimate ASM. A previous study showed a strong correlation between the ASM measured using this device and that measured by DXA (iDXA; GE Healthcare, Madison, WI, USA) (*R* ^2^ = 0.92).^15^ Given the device's non‐public proprietary formula, we also estimated ASM using the formula by Yamada *et al*.^16^ and performed a subanalysis (for results using this formula see Supporting Information).

We employed a DXA system (Delphi A [until December 2016] or Horizon A [after January 2017]; Hologic Inc., Marlborough, MA, USA) to measure body fat percentage and lean soft tissue mass. Inter‐instrument reliability of ASM between the two devices was excellent (intraclass correlation coefficient = 0.97). Participants were placed in a supine position on the DXA table for a whole‐body scan according to the manufacturer's protocol. The lean soft tissue mass of the entire body was divided into several areas, including the arms, legs and trunk. We calculated the ASM by adding up the lean soft tissue mass for the arms and legs. To adjust for build, we divided ASM (kg) by the square of the height (m^2^).

### *Definition of low muscle mass*

We defined LMM based on the AWGS 2019 recommended cut‐offs for muscle mass measurements.^6^ The cut‐offs for BIA‐measured ASM/height^2^ were 7.0 and 5.7 kg/m^2^ and those for DXA‐measured ASM/height^2^ were 7.0 and 5.4 kg/m^2^ for men and women, respectively.

### *Muscle strength measurement*

Hand‐grip strength was measured with a digital grip dynamometer (T.K.K.5401; Takei Scientific Instruments Co., Ltd., Niigata, Japan) twice for each hand. The average of the maximum values for each hand was calculated.

### *Statistical analysis*

We compared the mean values of the participants' characteristics between the non‐LMM and LMM groups measured by DXA using Student's *t*‐test. We calculated Pearson's correlation coefficients to evaluate the correlations between calf circumference and the BIA or DXA‐measured ASM/height^2^. We performed receiver operating characteristic (ROC) analysis to identify the optimal cut‐off for calf circumference in screening LMM measured by BIA and DXA. We calculated the areas under the ROC curve, 95% confidence intervals (95% CI), and the optimal cut‐off point, calculated by determining the shortest distance between the ROC curve and upper left corner of the graph. To examine the effect of obesity and age on the LMM screening measured by calf circumference, we divided the participants into two groups based on body fat percentage (non‐obese and obese) and age (middle‐aged: <60 years and older: ≥60 years) to perform subgroup analyses. Obesity was defined as a body fat percentage of ≥25% for men and ≥30% for women.^17^

Statistical significance was set at *P* < 0.05 in two‐tailed tests. All statistical analyses were performed using SPSS Statistics version 26 for Windows (IBM Corp., Armonk, NY, USA).

## Results

The mean ages for men and women were 57 (range 40–87) and 52 (40–84) years, respectively. The prevalence rates for LMM measured by BIA and DXA were 4.1% and 8.6% for men and 6.6% and 12.9% for women, respectively.

Table 1 shows the characteristics of the participants according to LMM measured by DXA. Participants with LMM exhibited significantly lower weight, body mass index (BMI), calf circumference, ASM, ASM/height^2^ and hand‐grip strength than those with non‐LMM, for both men and women. No significant differences were found in age, height and body fat percentage in the LMM and non‐LMM groups, for both men and women.

**Table 1:** Characteristics of the study participants according to low muscle mass measured by DXA in men and women

	Overall	Non‐low muscle mass	Low muscle mass^†^	*P* value
Men				
*n* (%)	827 (100.0)	756 (91.4)	71 (8.6)	
Age (years)	57 ± 10	57 ± 10	59 ± 12	0.114
Height (cm)	170.3 ± 5.8	170.4 ± 5.9	169.4 ± 5.1	0.178
Weight (kg)	69.0 ± 9.7	69.9 ± 9.5	59.5 ± 5.9	<0.001
Body mass index (kg/m^2^)	23.8 ± 3.0	24.1 ± 2.9	20.7 ± 1.7	<0.001
Calf circumference (cm)	37.6 ± 2.6	37.8 ± 2.5	34.5 ± 1.7	<0.001
Body fat by DXA (%)	20.4 ± 4.7	20.3 ± 4.7	21.3 ± 4.6	0.069
ASM by BIA (kg)	24.0 ± 3.1	24.4 ± 3.0	20.6 ± 1.8	<0.001
ASM by DXA (kg)	23.1 ± 3.0	23.5 ± 2.8	18.9 ± 1.4	<0.001
ASM/height^2^ by BIA (kg/m^2^)	8.3 ± 0.9	8.4 ± 0.8	7.2 ± 0.5	<0.001
ASM/height^2^ by DXA (kg/m^2^)	7.9 ± 0.8	8.1 ± 0.7	6.6 ± 0.3	<0.001
Hand‐grip strength (kg)	37.9 ± 5.8	38.4 ± 5.5	32.1 ± 6.0	<0.001
Women				
*n* (%)	412 (100.0)	359 (87.1)	53 (12.9)	
Age (years)	52 ± 9	52 ± 9	51 ± 7	0.174
Height (cm)	158.7 ± 5.3	158.5 ± 5.3	159.7 ± 5.2	0.118
Weight (kg)	53.8 ± 7.6	54.8 ± 7.5	47.6 ± 4.9	<0.001
Body mass index (kg/m^2^)	21.4 ± 2.9	21.8 ± 2.8	18.7 ± 1.5	<0.001
Calf circumference (cm)	34.4 ± 2.2	34.7 ± 2.0	32.1 ± 1.8	<0.001
Body fat by DXA (%)	27.2 ± 5.1	27.3 ± 5.2	26.8 ± 4.5	0.534
ASM by BIA (kg)	16.1 ± 1.7	16.3 ± 1.6	14.7 ± 1.1	<0.001
ASM by DXA (kg)	15.3 ± 2.0	15.7 ± 1.9	13.0 ± 1.0	<0.001
ASM/height^2^ by BIA (kg/m^2^)	6.4 ± 0.6	6.5 ± 0.5	5.8 ± 0.3	<0.001
ASM/height^2^ by DXA (kg/m^2^)	6.1 ± 0.7	6.2 ± 0.6	5.1 ± 0.2	<0.001
Hand‐grip strength (kg)	24.5 ± 3.7	24.8 ± 3.6	22.6 ± 3.6	<0.001

Data are expressed as mean ± standard deviation or *n* (%).

^†^ Low muscle mass was defined based on the Asian Working Group for Sarcopenia 2019‐recommended cut‐offs for muscle mass measurements measured by DXA (i.e., ASM/height^2^ <7.0 kg/m^2^ for men and <5.4 kg/m^2^ for women).

ASM, appendicular skeletal muscle mass; BIA, bioelectrical impedance analysis; DXA, dual‐energy X‐ray absorptiometry.

BIA‐measured ASM/height^2^ was positively correlated with DXA‐measured ASM/height^2^ (men: *r* = 0.88, women: *r* = 0.84; Fig. S2). We performed ROC analysis for screening LMM measured by DXA using BIA‐measured ASM/height^2^ (Fig. S3). The areas under the ROC curve for LMM measured by DXA were 0.92 (95% CI 0.90–0.95) for men and 0.90 (0.86–0.94) for women. The optimal cut‐offs for BIA‐measured ASM/height^2^ for screening LMM measured by DXA were 7.7 kg/m^2^ (sensitivity 87%, specificity 83%) for men and 5.9 kg/m^2^ (sensitivity 77%, specificity 89%) for women.

**Figure 1:**
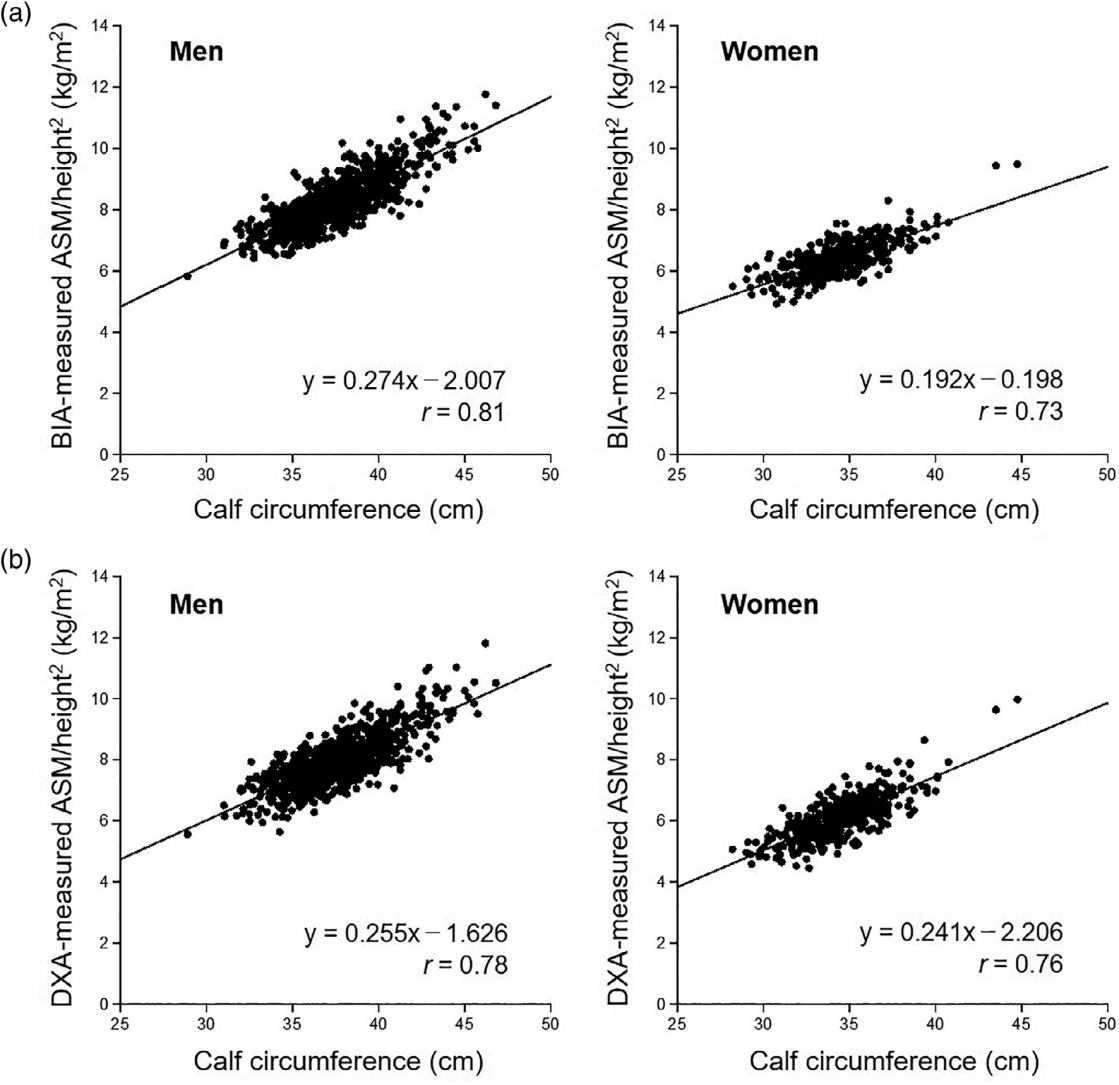
Correlation of calf circumference with (a) BIA and (b) DXA‐measured ASM/height^2^ in men and women. *r* = correlation coefficient. ASM, appendicular skeletal muscle mass; BIA, bioelectrical impedance analysis; DXA, dual‐energy X‐ray absorptiometry.

**Figure 2:**
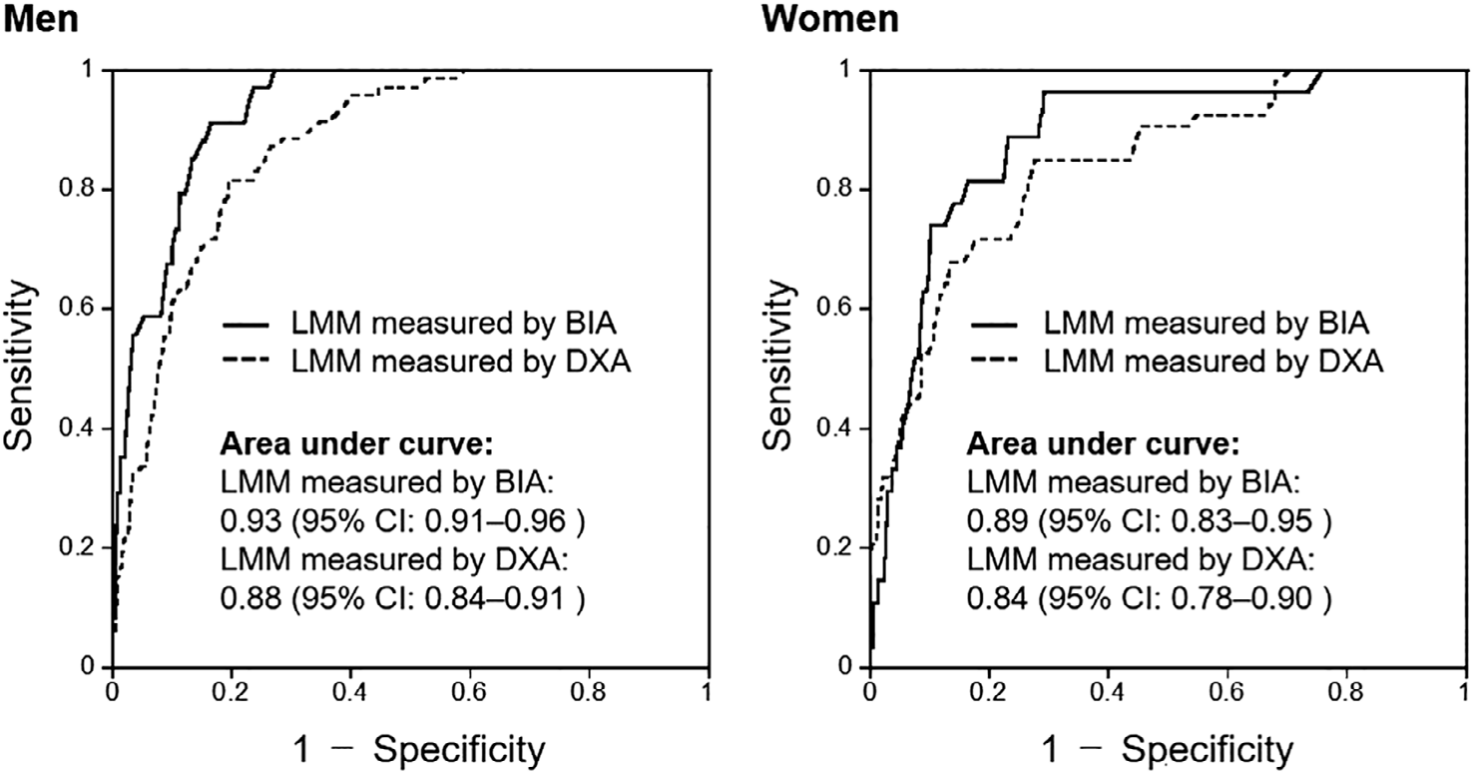
Receiver operating characteristic curves for screening LMM measured by BIA and DXA using calf circumference in men and women. BIA, bioelectrical impedance analysis; CI, confidence interval; DXA, dual‐energy X‐ray absorptiometry; LMM, low muscle mass.

Calf circumference was positively but weakly correlated with hand‐grip strength (men: *r* = 0.33, women: *r* = 0.31; Fig. S4). Figure 1 shows the correlations of calf circumference with BIA‐ and DXA‐measured ASM/height^2^. Calf circumference was positively correlated with BIA‐measured ASM/height^2^ (men: *r* = 0.81, women: *r* = 0.73) and DXA‐measured ASM/height^2^ (men: *r* = 0.78, women: *r* = 0.76). Table 2 and Fig. 2 show the results of ROC analysis for screening LMM using calf circumference values. The areas under the ROC curve for screening LMM measured by BIA and DXA were 0.93 (95% CI 0.91–0.96) and 0.88 (0.84–0.91) for men and 0.89 (0.83–0.95) and 0.84 (0.78–0.90) for women, respectively. The optimal calf circumference cut‐offs for screening LMM measured by BIA and DXA were 35.4 cm (sensitivity 91%, specificity 84%) and 35.8 cm (sensitivity 82%, specificity 80%) for men and 32.7 cm (sensitivity 82%, specificity 84%) and 33.5 cm (sensitivity 85%, specificity 72%) for women, respectively. We further calculated the calf circumference cut‐offs with maximum sensitivity or specificity (≥90%). The calf circumference cut‐offs for screening LMM measured by BIA and DXA with maximum sensitivity without excessively reducing specificity were 35.4 cm (sensitivity 91%, specificity 84%) and 36.7 cm (sensitivity 90%, specificity 67%) for men and 33.4 cm (sensitivity 93%, specificity 71%) and 34.4 cm (sensitivity 91%, specificity 55%) for women. Meanwhile, the cut‐offs with maximum specificity without excessively reducing sensitivity were 34.7 cm (sensitivity 71%, specificity 90%) and 35.0 cm (sensitivity 62%, specificity 90%) for men and 32.2 cm (sensitivity 74%, specificity 90%) and 32.4 cm (sensitivity 53%, specificity 90%) for women.

**Table 2:** Optimal calf circumference cut‐offs for screening low muscle mass in men and women

	Optimal calf circumference cut‐off (cm)	Sensitivity (%)	Specificity (%)
All men (*n* = 827)			
Low muscle mass measured by BIA^†^	35.4	91.2	83.5
Low muscle mass measured by DXA^‡^	35.8	81.7	80.4
Non‐obese men (*n* = 697)			
Low muscle mass measured by BIA^†^	35.2	85.7	85.4
Low muscle mass measured by DXA^‡^	35.8	78.6	78.0
Obese men (*n* = 130)			
Low muscle mass measured by BIA^†^	35.3	100.0	91.9
Low muscle mass measured by DXA^‡^	35.8	93.3	94.8
Middle‐aged men (*n* = 501)			
Low muscle mass measured by BIA^†^	35.4	92.3	86.9
Low muscle mass measured by DXA^‡^	36.3	84.2	78.6
Older men (*n* = 326)			
Low muscle mass measured by BIA^†^	35.3	90.5	81.0
Low muscle mass measured by DXA^‡^	35.8	87.9	73.4
All women (*n* = 412)			
Low muscle mass measured by BIA^†^	32.7	81.5	83.6
Low muscle mass measured by DXA^‡^	33.5	84.9	72.4
Non‐obese women (*n* = 286)			
Low muscle mass measured by BIA^†^	32.2	85.7	87.2
Low muscle mass measured by DXA^‡^	32.9	73.2	77.6
Obese women (*n* = 126)			
Low muscle mass measured by BIA^†^	33.5	100.0	84.2
Low muscle mass measured by DXA^‡^	33.5	75.0	84.2
Middle‐aged women (*n* = 325)			
Low muscle mass measured by BIA^†^	32.7	84.2	83.3
Low muscle mass measured by DXA^‡^	33.5	85.4	74.7
Older women (*n* = 87)			
Low muscle mass measured by BIA^†^	33.1	100.0	77.2
Low muscle mass measured by DXA^‡^	31.8	60.0	93.9

^†^ Low muscle mass was defined based on the Asian Working Group for Sarcopenia 2019‐recommended cut‐offs for muscle mass measurements measured by BIA (i.e., ASM/height^2^ <7.0 kg/m^2^ for men and <5.7 kg/m^2^ for women).

^‡^ Low muscle mass was defined based on the Asian Working Group for Sarcopenia 2019‐recommended cut‐off points for muscle mass measurements measured by DXA (i.e., ASM/height^2^ <7.0 kg/m^2^ for men and <5.4 kg/m^2^ for women).

Obesity was defined as a body fat percentage measured by DXA ≥25% for men and ≥30% for women. Participants were divided into two groups: middle‐aged adults (age <60 years) and older adults (age ≥60 years).

ASM, appendicular skeletal muscle mass; BIA, bioelectrical impedance analysis; DXA, dual‐energy X‐ray absorptiometry.

We also performed a subanalysis using the publicly available ASM estimation formula for BIA (for results see Supporting Information). Although the correlation between calf circumference and BIA‐measured ASM/height^2^ was slightly weaker, a positive correlation was found similar to that shown in the main analysis (men: *r* = 0.68, women: *r* = 0.66; Fig. S5). The areas under the ROC curve and optimal calf circumference cut‐offs for LMM were similar to the results of the main analysis (Fig. S6).

**Figure 3:**
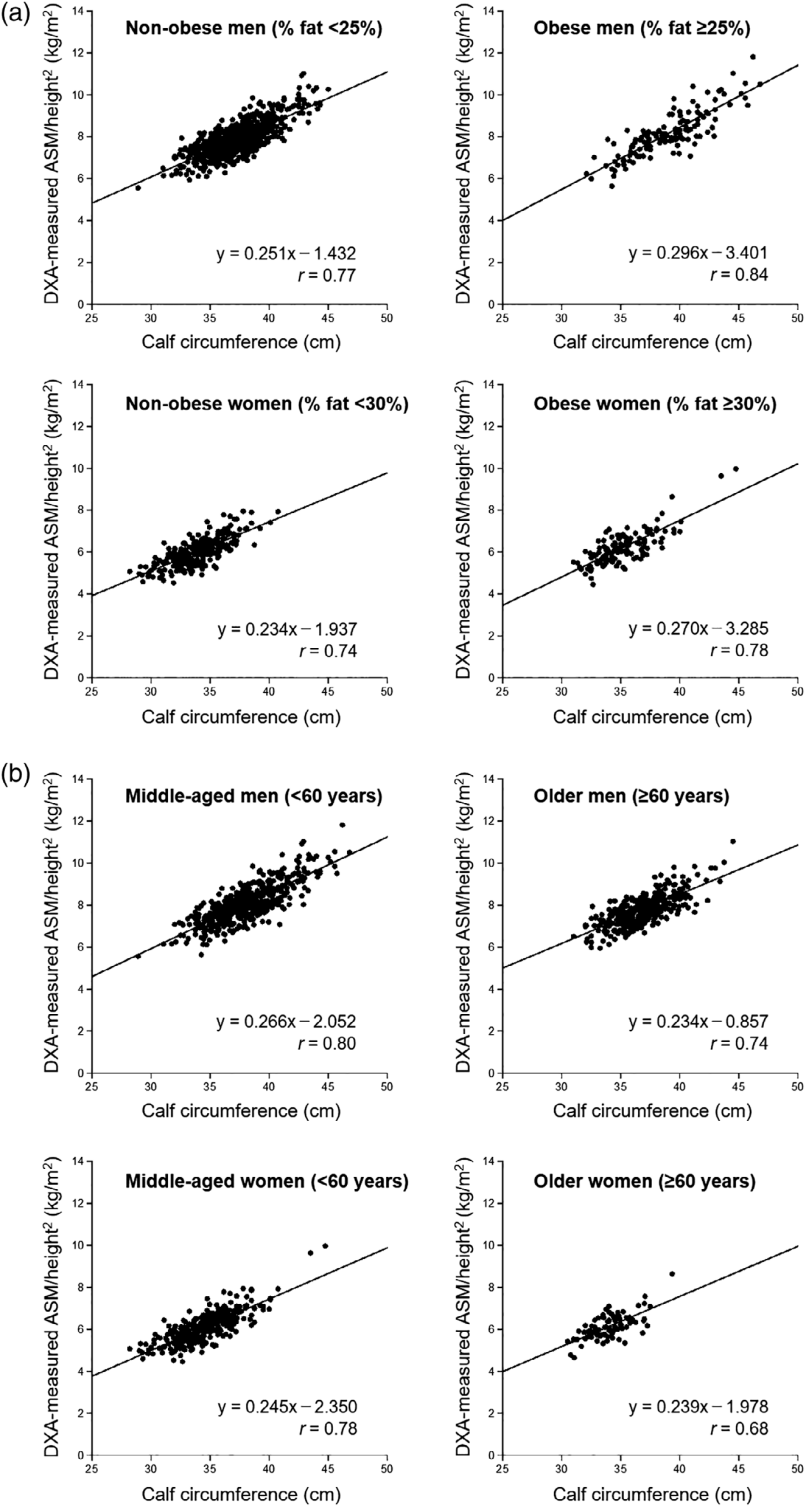
Correlation between calf circumference and DXA‐measured ASM/height^2^ according to (a) obesity and (b) age in men and women. *r* = correlation coefficient. ASM, appendicular skeletal muscle mass; DXA, dual‐energy X‐ray absorptiometry.

We also did an analysis according to subgroups that were based on obesity and age. The obesity prevalence rates were 15.7% for men and 30.6% for women. The proportions of older adults were 39.4% for men and 21.1% for women. Calf circumference was positively correlated with DXA‐measured ASM/height^2^ regardless of obesity and age (Fig. 3). The optimal calf circumference cut‐offs for screening LMM measured by BIA and DXA were approximately similar to those of the main analysis (Table 2).

## Discussion

We investigated the association of calf circumference with ASM/height^2^ and confirmed the validity of optimal calf circumference cut‐offs in LMM screening. We also examined the effect of obesity and age on LMM screening measured by calf circumference. Calf circumference was positively correlated with BIA‐measured ASM/height^2^ (men: *r* = 0.81, women: *r* = 0.73) and DXA‐measured ASM/height^2^ (men: *r* = 0.78, women: *r* = 0.76). In the subgroup analyses by obesity and age, calf circumference was also positively correlated with DXA‐measured ASM/height^2^.

Previous studies that examined human cadavers, such as the study involving male cadavers (*n* = 12) in Brussels^18^ and the study involving white cadavers (*n* = 23),^19^ reported that the maximal calf circumference was positively correlated with total dissected muscle mass (*r* = 0.84, or men: *r* = 0.90 and women: *r* = 0.77). Furthermore, maximal calf circumference was strongly correlated with calf muscle mass measured by magnetic resonance imaging (men: *r* = 0.91, women: *r* = 0.89).^20^ Calf circumference was positively correlated with muscle mass measured by BIA^21^, ^22^, ^23^ or DXA^7^, ^8^, ^9^, ^10^, ^11^ (BIA‐measured ASM: *r* = 0.68, BIA‐measured ASM/height^2^: *r* = 0.56–0.78, DXA‐measured ASM: *r* = 0.55–0.81, DXA‐measured ASM/height^2^: *r* = 0.42–0.84). Moreover, the National Health and Nutrition Examination Survey that examined 15 293 participants reported a moderate correlation between calf circumference and DXA‐measured ASM across all BMI subgroups (<18.5, 18.5–24.9, 25–29.9, ≥30 kg/m^2^) or age subgroups (<20, 20–39, 40–59, ≥60 years).^12^ Our results are consistent with those of previous studies, suggesting that calf circumference is positively correlated with muscle mass regardless of obesity and age and can be employed for LMM screening measured by BIA and DXA.

Our study calculated the optimal calf circumference cut‐offs for LMM screening measured by BIA and DXA: 35 cm (sensitivity 91%, specificity 84%) and 36 cm (sensitivity 82%, specificity 80%) for men and 33 cm (sensitivity 82%, specificity 84%) and 34 cm (sensitivity 85%, specificity 72%) for women. The cut‐offs were similar between BIA and DXA. We also performed the subgroup analyses based on obesity and age to calculate optimal cut‐offs, which were roughly similar to the main results. A study on hospitalized patients found no difference in calf circumference between those with normal weight and those who were overweight or obese, as assessed by BMI.^24^ Calf circumference appears to be less susceptible to body fat, and our results suggest that obesity does not affect muscle mass prediction based on calf circumference. Multifrequency BIA or DXA use is recommended by the AWGS 2019 consensus for assessing muscle mass in diagnosing sarcopenia.^6^ Previous studies examined the optimal maximal calf circumference cut‐offs in LMM screening as measured by multifrequency BIA and DXA (Table S1). If we limit the scope to studies conducted in Asian countries, there are five studies conducted in Taiwan, Korea, Indonesia and Japan, to the best of our knowledge.^7^, ^8^, ^11^, ^13^, ^14^ Although all these studies employed the same muscle mass cut‐off proposed by the AWGS for determining LMM, the posture in which the calf circumference was measured ranged from supine, sitting, to standing. Studies that examined elderly individuals^25^ and hospitalized patients^24^ reported that calf circumference measurements in the supine position are smaller by 0.5–0.6 cm on average than those measured in the standing position. In fact, low cut‐offs have been reported in studies that investigated calf circumference, measured in the supine position.^9^, ^14^ Further research that considers the posture in which measurements are obtained is required for those cases in which LMM is assessed by measuring the calf circumference in the supine position in hospitals and nursing homes. Only three studies conducted in Asia measured calf circumference in the standing position, and the optimal calf circumference cut‐offs were 34–35 cm (sensitivity 65–92%, specificity 59–88%) for men and 29–33 cm (sensitivity 71–83%, specificity 50–96%) for women.^7^, ^8^, ^11^ Although it should be noted that different ASM/height^2^ cut‐offs were employed for determining LMM, four studies conducted in Brazil, France and South Africa revealed optimal calf circumference cut‐offs of 34 cm (sensitivity 61–71%, specificity 76–77%) for men and 30–33 cm (sensitivity 80–100%, specificity 76–93%) for women, similar to those reported in studies that examined Asian individuals, except for one study where calf circumference was measured in the supine position.^9^, ^10^, ^26^, ^27^ The results of our study and those of previous studies suggest that maximum calf circumference is a simple and accurate surrogate marker for finding cases of LMM as assessed by BIA or DXA. However, criteria should be established for optimal cut‐offs that account for measurement conditions, such as posture and edema, given that few studies examined optimal calf circumference cut‐offs, and measurement conditions such as posture can vary.

To date, several studies have determined the cut‐offs for LMM screening measured by calf circumference (Table S1). However, to the best of our knowledge, our study is the first to employ and compare both BIA and DXA. Furthermore, we performed an analysis that considered obesity and age, and demonstrated for the first time that LMM can be screened by calf circumference regardless of obesity and age. Our study has several limitations. First, the participants were Waseda University alumni and their spouses who opted to participate and were not randomly selected from the population. Therefore, the sample may not have been representative of the general population. In addition, the number of older women in our study was particularly small. Further studies with participants of a wider age range are required. Second, we did not consider the effect of edema that reduces the accuracy of muscle mass estimation using calf circumference measurements although calf circumference was measured in the morning in our study. Pitting edema increases calf circumference by 2.0 cm in men and 1.6 cm in women.^28^ Overestimation of muscle mass should be considered in those with edema. Third, given that this was a cross‐sectional study, further research is required to confirm whether changes in muscle mass can be estimated by calf circumference. Although a study that involved a 3‐month exercise intervention reported an increase in BIA‐measured muscle mass and calf circumference after the intervention,^29^ a longitudinal study reported that calf circumference was a predictor of the onset of sarcopenia.^30^

In conclusion, calf circumference is positively correlated with BIA‐ and DXA‐measured ASM/height^2^ regardless of obesity and age, and is a simple and accurate surrogate marker of muscle mass for diagnosing sarcopenia measured by both BIA and DXA.

## Disclosure statement

The authors declare no conflict of interest.

## Supporting information


**Figure S1.** Method of calf circumference measurement.
**Figure S2.** Correlation between BIA‐measured ASM/height^2^ and DXA‐measured ASM/height^2^ in men and women.
**Figure S3.** Receiver operating characteristic curves for screening low muscle mass measured by DXA using BIA‐measured ASM/height^2^ in men and women.
**Figure S4.** Correlation between calf circumference and hand‐grip strength in men and women.
**Figure S5.** Correlation between calf circumference and BIA‐measured ASM/height^2^ estimated by Yamada *et al*. formula in men and women.
**Figure S6.** Receiver operating characteristic curves for screening low muscle mass measured by BIA estimated by Yamada *et al*. formula using calf circumference in men and women.
**Table S1.** Previous studies examining the calf circumference cut‐offs for screening low muscle mass measured by multifrequency BIA and DXA.
