# Supplementary material for: Cut‐offs for calf circumference as a screening tool for low muscle mass: WASEDA'S Health Study
Source: Geriatr Gerontol Int. 2020 Sep 4;20(10):943–50. doi: 10.1111/ggi.14025 (PMC7590124; doi:10.1111/ggi.14025)
Supplement: Supplementary file 1 — Figure S1. Method of calf circumference measurement. Figure S2. Correlation between BIA‐measured ASM/height2 and DXA‐measured ASM/height2 in men and women. Figure S3. Receiver operating characteristic curves for screening low muscle mass measured by DXA using BIA‐measured ASM/height2 in men and women. Figure S4. Correlation between calf circumference and hand‐grip strength in men and women. Figure S5. Correlation between calf circumference and BIA‐measured ASM/height2 estimated by Yamada et al. formula in men and women. Figure S6. Receiver operating characteristic curves for screening low muscle mass measured by BIA estimated by Yamada et al. formula using calf circumference in men and women. Table S1. Previous studies examining the calf circumference cut‐offs for screening low muscle mass measured by multifrequency BIA and DXA. [file GGI-20-943-s001.docx]

**Figure S1** Method of calf circumference measurement. The maximal calf circumference was measured to the nearest 0.1 cm with the use of a steel measuring tape while the participant was in the standing position, without the compression of the subcutaneous tissue.


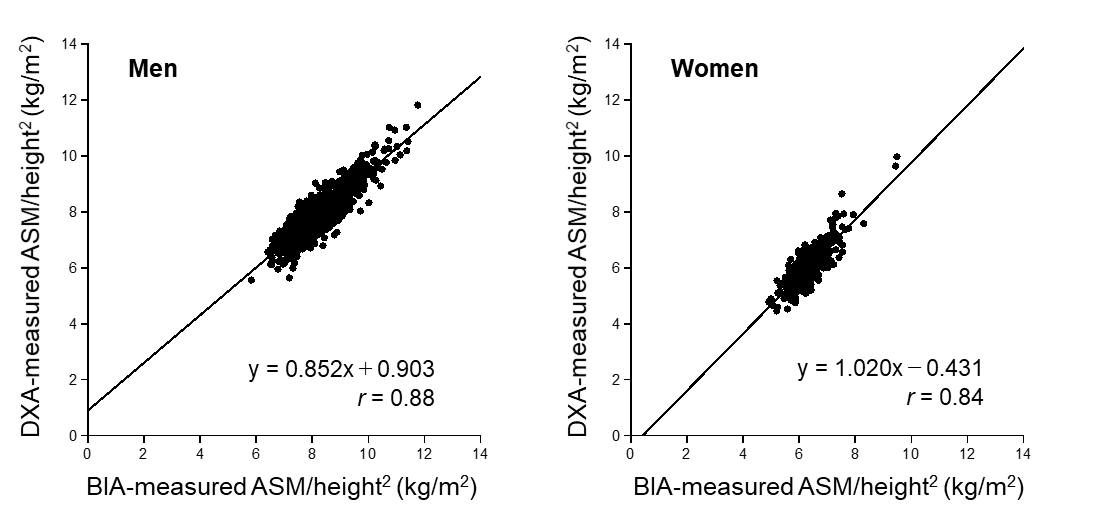


**Figure S2** Correlation between BIA-measured ASM/height^2^ and DXA-measured ASM/height^2^ in men and women. *r* = correlation coefficient. ASM, appendicular skeletal muscle mass; BIA, bioelectrical impedance analysis; DXA, dual-energy X-ray absorptiometry.

**
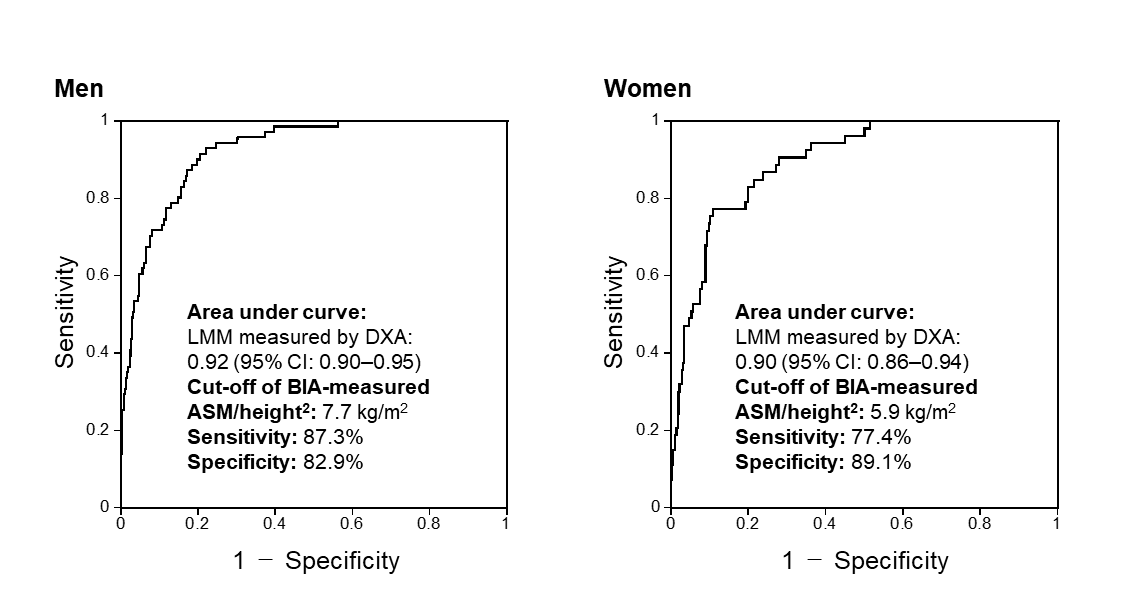
**

**Figure S3** Receiver operating characteristic curves for screening low muscle mass measured by DXA using BIA-measured ASM/height^2^ in men and women. ASM, appendicular skeletal muscle mass; BIA, bioelectrical impedance analysis; CI, confidence interval; DXA, dual-energy X-ray absorptiometry; LMM, low muscle mass.

**
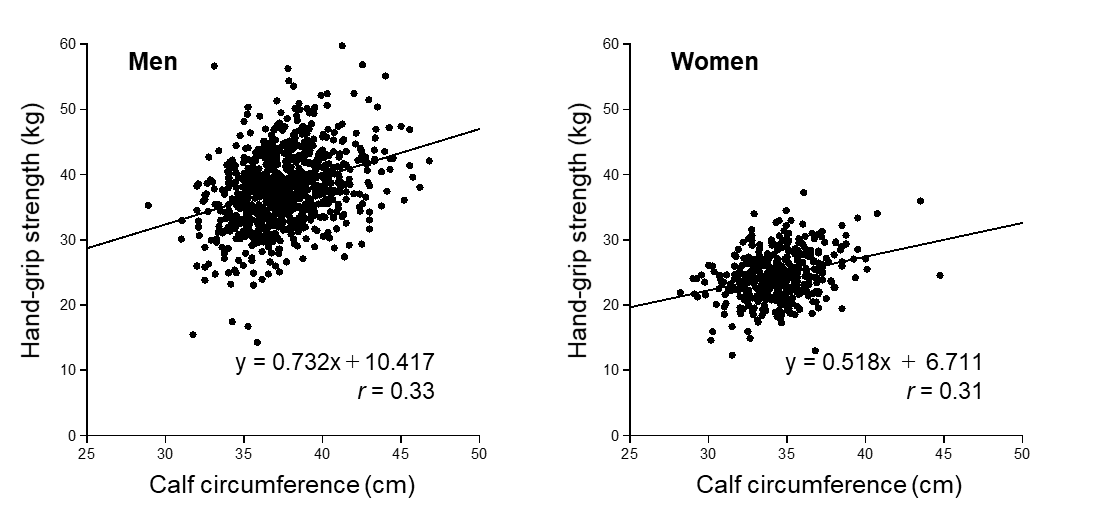
**

**Figure S4** Correlation between calf circumference and hand-grip strength in men and women. *r* = correlation coefficient.

**
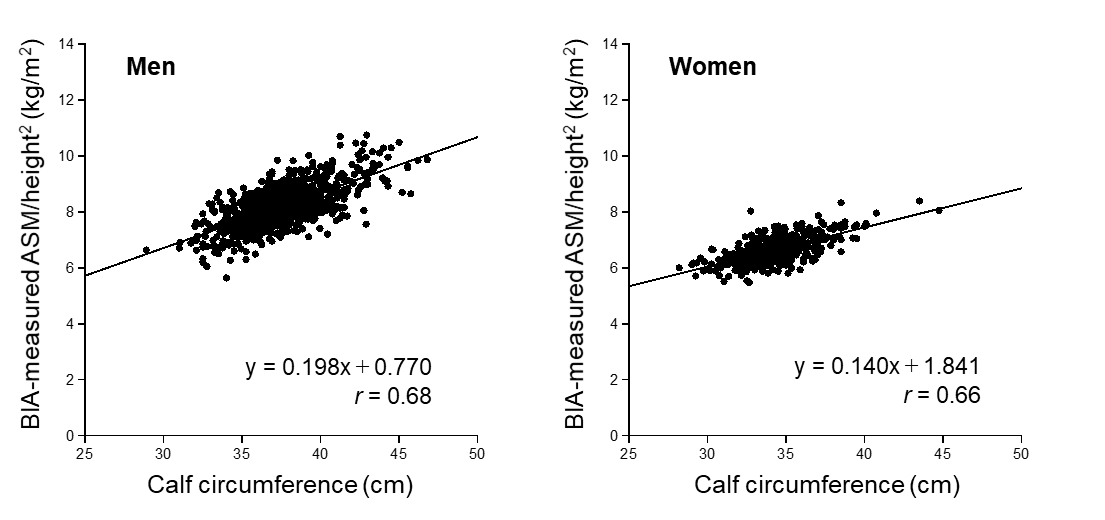
**

**Figure S5** Correlation between calf circumference and BIA-measured ASM/height^2^ estimated by Yamada *et al*. formula in men and women. *r* = correlation coefficient. ASM, appendicular skeletal muscle mass; BIA, bioelectrical impedance analysis. ASM = (0.6947 × (height^2^/Z_50_)) + (−55.24 × (Z_250_/Z_5_)) + (−10940 × (1/Z_50_)) + 51.33 for men, and ASM = (0.6144 × (height^2^/Z_50_)) + (−36.61 × (Z_250_/Z_5_)) + (−9332 × (1/Z_50_)) + 37.91 for women.

Reference: Yamada Y, Nishizawa M, Uchiyama T, *et al*. Developing and validating an age-independent equation using multi-frequency bioelectrical impedance analysis for estimation of appendicular skeletal muscle mass and establishing a cutoff for sarcopenia. *Int J Environ Res Public Health* 2017; 14: E809.

**
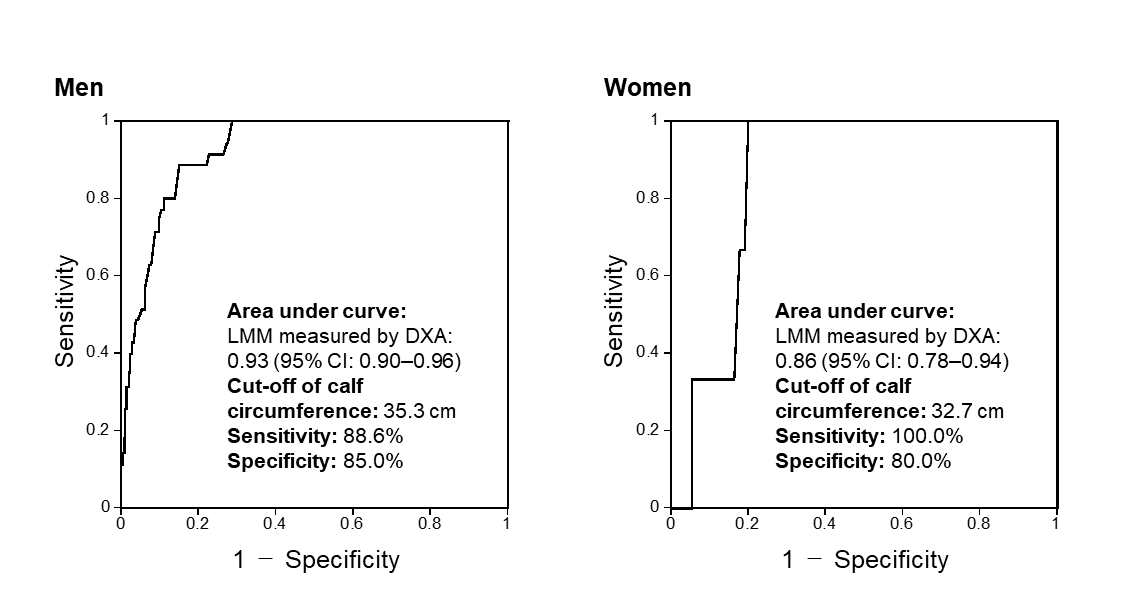
**

**Figure S6** Receiver operating characteristic curves for screening low muscle mass measured by BIA estimated by Yamada *et al*. formula using calf circumference in men and women. ASM, appendicular skeletal muscle mass; BIA, bioelectrical impedance analysis; CI, confidence interval; DXA, dual-energy X-ray absorptiometry; LMM, low muscle mass. ASM = (0.6947 × (height^2^/Z_50_)) + (−55.24 × (Z_250_/Z_5_)) + (−10940 × (1/Z_50_)) + 51.33 for men, and ASM = (0.6144 × (height^2^/Z_50_)) + (−36.61 × (Z_250_/Z_5_)) + (−9332 × (1/Z_50_)) + 37.91 for women.

Reference: Yamada Y, Nishizawa M, Uchiyama T, *et al*. Developing and validating an age-independent equation using multi-frequency bioelectrical impedance analysis for estimation of appendicular skeletal muscle mass and establishing a cutoff for sarcopenia. *Int J Environ Res Public Health* 2017; 14: E809.

**Table S1** Previous studies examining the calf circumference cut-offs for screening low muscle mass measured by multifrequency BIA and DXA

| Author (year) | Country | *n* | Mean age (range) | Muscle mass measurement | Position of CC measurement | Sex | Diagnostic criteria | CC cut-off (cm) | Sensitivity (%) | Specificity (%) | AUC  (95% CI) |
| --- | --- | --- | --- | --- | --- | --- | --- | --- | --- | --- | --- |
| **Asian countries** | | | | | | | | | | | |
| Hwang AC *et al*. (2018) | Taiwan | 1839 | 63.9  (50–92) | DXA (GE) | Sitting | Men | ASM/height^2^ 7.0 kg/m^2^ | 33 | 82 | 66 | 0.81  (0.77–0.86) |
|  |  |  |  |  |  | Women | ASM/height^2^ 5.4 kg/m^2^ | 32 | 74 | 63 | 0.75  (0.69–0.80) |
| Kim S *et al*. (2018) | Korea | 657 | 76.2  (70–84) | DXA (GE) | Standing | Men | ASM/height^2^ 7.0 kg/m^2^ | 35 | 92 | 59 | 0.81  (0.76–0.86) |
|  |  |  |  |  |  | Women | ASM/height^2^ 5.4 kg/m^2^ | 33 | 83 | 50 | 0.72  (0.66–0.79) |
| Laksmi PW *et al*. (2019)^†^ | Indonesia | 120 | 71.9  (60–) | DXA | Standing | Men | ASM/height^2^ 7.0 kg/m^2^ | 34 | 65 | 79 | 0.73  (0.57–0.89) |
|  |  |  |  |  |  | Women | ASM/height^2^ 5.4 kg/m^2^ | 29 | 71 | 96 | 0.96  (0.92–1.00) |
| Kawakami R *et al*. (2015) | Japan | 526 | 61.2  (40–89) | DXA (Hologic) | Standing | Men | ASM/height^2^ 7.0 kg/m^2^ | 34 | 89 | 88 | 0.94  (0.89–1.00) |
|  |  |  |  |  |  | Women | ASM/height^2^ 5.4 kg/m^2^ | 33 | 78 | 72 | 0.84  (0.77–0.92) |
| Maeda K *et al*. (2017)^‡^ | Japan | 1164 | 83.5  (65–) | BIA (InBody) | Supine | Men | ASM/height^2^ 7.0 kg/m^2^ | 31 | 75 | 75 | 0.83  (0.79–0.88) |
|  |  |  |  |  |  | Women | ASM/height^2^ 5.7 kg/m^2^ | 29 | 78 | 70 | 0.79  (0.74–0.85) |
| **The other countries** | | | | | | | | | | | |
| Pagotto V *et al*. (2018) | Brazil | 132 | 70.2  (60–91) | DXA (GE) | Standing | Men | ASM/height^2^ 7.26 kg/m^2^ | 34 | 71 | 77 | 0.81  (0.69–0.93) |
|  |  |  |  |  |  | Women | ASM/height^2^ 5.45 kg/m^2^ | 33 | 80 | 85 | 0.90  (0.83–0.98) |
| Barbosa-Silva TG *et al*. (2016) | Brazil | 189 | Median 69 (60–90) | DXA (GE) | Standing | Men | ASM/height^2^ 7.76 kg/m^2^ | 34 | 61 | 76 | 0.76  (0.64–0.88) |
|  |  |  |  |  |  | Women | ASM/height^2^ 5.62 kg/m^2^ | 33 | 100 | 76 | 0.91  (0.84–0.97) |
| Ukegbu PO et al. (2018) | South Africa | 247 | Median 56 (45–) | DXA (Hologic) | Standing | Women | ASM/height^2^ 4.94 kg/m^2^ | 30 | 100 | 93 | 0.94  (0.96–1.00) |
| Rolland Y *et al*. (2003) | France | 1458 | 80.3  (70–) | DXA (Hologic) | Supine | Women | ASM/height^2^ 5.45 kg/m^2^ | 31 | 44 | 91 | N/A |

ASM, appendicular skeletal muscle mass; AUC, area under ROC curve; BIA, bioelectrical impedance analysis; CC, calf circumference; CI, confidence interval; DXA, dual-energy X-ray absorptiometry; N/A, not available.

We contacted the authors directly, where possible, regarding the data for items not reported in the studies.

^†^The study included outpatients from the Geriatric Clinic.

^‡^The study included consecutive patients, who stayed in the hospital for ≥5 days.
